# Academic stress and irregular menstruation influence the dysmenorrhea, school absenteeism and healthcare seeking among adolescent girls in junior high school in Shanghai: a cross-sectional study

**DOI:** 10.3389/frph.2025.1574195

**Published:** 2025-07-18

**Authors:** Tiantian Liu, Deyun Qi, Li Zhang, Jun Hou, Jing Zhao, Yuan Zhou, Bingbing Sun, Fei Wang, Hui Tan, Ruiping Wang

**Affiliations:** ^1^ Department of Children’s Eye Disease Prevention, Hongkou Center for Disease Control and Prevention, Shanghai, China; ^2^ Center of Disease Prevention and Control for Mental Disorders, Jing’an District Mental Health Center, Shanghai, China; ^3^ Hongkou District Guangzhong Road Street Community Health Service Center, Shanghai, China; ^4^ Hongkou Center for Disease Control and Prevention, Shanghai, China; ^5^ Key Laboratory of Public Health Safety, Ministry of Education, School of Public Health, Fudan University, Shanghai, China; ^6^ Clinical Research Center, Shanghai Skin Diseases Hospital, School of Medicine, Tongji University, Shanghai, China

**Keywords:** adolescent girls, dysmenorrhea, academic stress, school absenteeism, healthcare seeking behavior, junior high school

## Abstract

**Background:**

Dysmenorrhea is a cramping pain during menstrual period and the leading cause for recurrent short-term school absenteeism among adolescent girls. However, evidence on the factors associated with the occurrence of dysmenorrhea, school absenteeism and health care seeking behavior among adolescent girls is still limited, especially in China. This study aim to understand the prevalence of dysmenorrhea and to explore factors associated with the school absenteeism and healthcare seeking habit among adolescent girls with dysmenorrhea.

**Methods:**

This cross-sectional,questionnaire-based study included 1,243 participants recruited from five junior high schools using cluster sampling method. Data were collected through a structured questionnaire interviews and SPSS 22.0 was used for data analysis.

**Results:**

The prevalence of dysmenorrhea was 67.2%. Logistic regression analysis indicated that adolescent girls in grade 8 (OR = 1.98, 95% CI: 1.32–2.97) and grade 9 (OR = 2.32, 95% CI: 1.54–3.48), whose mothers had a college and above education (OR = 1.85; 95% CI: 1.12–3.07), those with controllable learning burden (OR = 1.69, 95% CI: 1.10–2.60) and uncontrollable learning burden (OR = 2.04, 95% CI: 1.10–3.80) had higher prevalence of dysmenorrhea. Logistic regression indicated that adolescent girls with normal weight (OR = 0.60, 95% CI: 0.43–0.83) had lower proportion of school absenteeism, and adolescent girls with uncontrollable learning burden (OR = 2.73, 95% CI: 1.29–5.75) and with irregular menstruation (OR = 1.74, 95% CI: 1.26–2.39) had higher proportion of school absenteeism. Moreover, underweight adolescent girls, adolescent girls whose mother had senior high education and those with irregular menstruation had a higher proportion of healthcare seeking experience.

**Conclusions:**

Dysmenorrhea was common among adolescent girls in junior high schools in Shanghai, and academic stress as well as irregular menstruation was positively associated with dysmenorrhea and school absenteeism. More attention and intervention measures focusing on menstrual health problems should be implemented directly among adolescent girls in junior high school, especially among those with low body weight, irregular menstruation, and academic stress. Most importantly, this study provides scientific evidence on adolescent dysmenorrhea issues, offering targeted recommendations for policymakers to advance the refinement and implementation of public health policies.

## Introduction

Dysmenorrhea is a cramping pain that occurs during woman's menstrual period, mostly in the lower abdomen. Dysmenorrhea can be classified as two categories: primary and secondary dysmenorrhea (1–3). Primary dysmenorrhea has no organic lesions, but secondary dysmenorrhea has conspicuous organic pathologies like endometriosis and pelvic inflammatory disease (4, 5). Dysmenorrhea is one of the most frequently happened gynaecological disorders among adolescent girls, and it causes a serious diseases burden than any other gynecological complaint (6–8). Previous studies have indicated that dysmenorrhea substantially contributes to the quality-adjusted life year loss in reproductive age group, and also causes significant economic losses (9, 10). Dysmenorrhea has a widespread impact on daily activities, personal relationships, academic performance, recurrent short term school and works absenteeism, and physical well-being, with longstanding impairments on quality of life and life course potential (7, 11, 12).Generally, dysmenorrhea is treated as an important public health issue worldwide.

The prevalence of dysmenorrhea among adolescents and young women varies from 45% to 95% among females with different age as well as different assessment tools (13, 14). A systematic review encompassing 21,573 young women across countries with varying income levels revealed that the prevalence of dysmenorrhea was as high as 71.1% (15). Recent epidemiological studies have documented significant variations in the global prevalence of dysmenorrhea, with reported rates of 71.69% among Ethiopian women (16), 57.9% in Spanish populations (17), 56% in Brazilian cohorts (18), and remarkably high prevalence reaching 85% in Dutch samples (19). In comparison, Chinese adolescents exhibit a relatively lower prevalence of 41.77% according to recent national surveys (20). Dysmenorrhea affects the recurrent short-term school and work absenteeism, and limit academic, social and physical activities in adolescent girls (13, 21). Previous studies show that more than one-half of patients with dysmenorrhea had encountered social withdrawal and decrease in academic performance (22). Previous studies indicated that 20% of adolescent girls had school absenteeism and over 40% of them reported learning performance or classroom concentration was negatively affected (15). Studies show that age at menarche, body mass index (BMI), family history, menstrual flow, lifestyle, anxiety, depression and stress are associated with dysmenorrhea in adolescent girls (1, 16, 20, 23). Academic stress among junior high school students poses an obvious challenge to their daily lifestyle, therefore, academic stress is probably an important factor associated with the occurrence of dysmenorrhea.

Despite of the availability of effective, easy-to-use and accessible therapeutic methods, most girls do not seek the healthcare for dysmenorrhea and only very few adolescent girls accept pharmacological treatment (24). A study in Australia shows that among young women, the proportion of those who talk to doctors is slightly lower than one-third (31.1%) (25), which is comparable to a study in Hunan, China (27.4%) (26). Previous studies indicate that almost 14% of Hispanic female adolescents with dysmenorrhea do not seek health care (27), which was in line with the conditions in Japan (15%) (28) and Hong Kong (6.4%) (29), and there are various reasons for not seeking health care (30). Previous studies indicated that many females consider the pain is a normal part of the menstrual cycle rather than a disorder (31) and lower proportion of healthcare seeking may lead to the increased diagnostic and treatment delays for women with dysmenorrhea symptoms. Thus, it is important for adolescents girls with dysmenorrhea to receive healthcare service to improve their quality of life.

As previously mentioned, dysmenorrhea is the most common gynecological problem among adolescent girls. However, evidence on the association between academic stress and the occurrence of dysmenorrhea, as well as factors associated with the school absenteeism and healthcare seeking habit due to dysmenorrhea in China is still limited. In this cross sectional study, we aimed to investigate the prevalence and risk factors of dysmenorrhea among adolescent girls in junior high schools in Shanghai, China, and to explore factors associated with the school absenteeism and lower healthcare seeking habit. Moreover, the findings furnish scientific substantiation for refining and enforcing public health policies aimed at enhancing the health and well-being of adolescent girls.

## Materials and methods

### Study population

This study used a cluster sampling method to recruit adolescent girls in junior high schools from October 2023 to November 2023. First, 5 out of the total 18 junior high schools were randomly selected in Hongkou district of Shanghai (We conducted a survey on the scale of the schools in the early stage and found that the number of female students in each school was approximately 300. For this study, we determined the required sample size to be 1,242 based on the sample size calculation formula. Considering the scale of the schools and the convenience of communication, we finally selected five schools to implement this research.). Second, all adolescent girls in the 5 selected junior high schools who satisfied the inclusion criteria were surveyed with informed consent. The inclusion criteria were as follows: (1) adolescent girls aged 11–15 years; (2) grade 6 to grade 9 in junior high school; (3) voluntarily participated in the study, read and signed informed consent. Adolescent girls with communication barriers or with incomplete questionnaire filling were excluded. This study was reviewed and approved by the Ethics Committee of the School of Public Health, Fudan University (IRB#2022-11-1010), the informed consent was obtained from each adolescent girls as well as their parents or legal guardians for those under the age of 16 before starting the study, and the implementation of this study was strictly adhere to the declaration of Helsinki.

### Sample size

In this study, sample size was calculated using the formula for cross-sectional study $n=\frac{Z_{\alpha }^{2}P(1-P)}{\delta^{2}}$, where Z_α_ = 1.96 when *α*=0.05, *P* is the prevalence of dysmenorrhea among adolescent girls in junior high school (approximately 65.5% in a previous study (32) and δ is admissible error. In this study, we set *α*=0.05, the prevalence of dysmenorrhea (*P*) was 65%, and *δ*=5% of *P*, the sample size was 828, considering a 10% of non-response rate and a 80% prevalence of menstruation in adolescent girls in junior high schools, at least 1,242 adolescent girls should be recruited. In this study, 1,243 adolescent girls in junior high schools in Hongkou district, Shanghai were finally recruited and analyzed in this study.

### Measures

**Figure 1:**
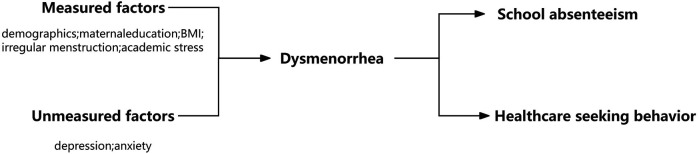
Conceptual model for the proposed relationships between the study variables and dysmenorrhea and school absenteeism and health seeking behavior.

In this study, the conceptual model is depicted in Figure 1, which visually represents the hypothesized relationships between our study variables based on current literature.

### Data collection

In this study, a self-designed questionnaire aligned with the definition of menstrual health was used for data collection through face to face interviews. The questionnaire consisted of: (1) demographic features, including birth date, height, weight, ethnicity, education of the mother and father, and family income etc.; (2) academic stress covered three options: without learning burden, with controllable learning burden, and with uncontrollable learning burden; (3) menstrual characteristics, including age at first menarche, menstrual history in the last year (menstrual cycle length, cycle regularity, menstrual length, menstrual blood volume, feature of dysmenorrhea, etc.), school absenteeism, and healthcare seeking experience due to menstrual problems.

### Definitions and classifications

In this study, body mass index (BMI) was calculated as weight in kilograms divided by the height in meters squared, and was classified into low weight (<18.5), normal weight (18.5-23.9), and overweight or obesity (≥24.0). Family income was divided into higher level (>1.0 million RMB/year), moderate level (0.2–1.0 million RMB/year), and lower level (<0.2 million RMB/year). Education of parents was recorded as the number of years of schooling completed and was categorized as 0–9 years (junior high school and lower), 10–12 years (senior high school), and >12 years (college and above), and no response as well. School absenteeism refers to the proportion of students with dysmenorrhea who are absent from school due to dysmenorrhea. Healthcare seeking experience refers to the proportion of students with dysmenorrhea who seek professional medical care for their condition. In this study, we defined irregular menstruation as an irregular menstrual cycle with a difference of ≥7 days between short and long cycles in the past year. NRS (numeric rating scale) is the frequently used pain scale and is regarded as a valid instrument for evaluating menstrual pain evaluation. In this study, we assessed the severity of dysmenorrhea by the numeric rating scale (NRS) with scores ranging from 0 to 10, and categorized into no pain (0), mild (1–3), moderate (4–6), and severe (7–10) (33).

### Statistical analysis

In this study, data analysis was performed using SPSS version 22.0 software (IBM Corp). Quantitative variables were described as the mean and standard deviation (SD) or median and interquartile ranges (IQR) as appropriate. We applied student's *t*-test, analysis of variance (ANOVA) or rank-sum test to examine difference between groups for quantitative variables. Qualitative variables were described as frequency counts and proportion (%), and the chi-square test was used to examine difference between groups. Logistic regression model was used to calculate the odds ratios (OR) and 95% confidence intervals (CI) to explore the influencing factors associated with dysmenorrhea, school absenteeism, and healthcare seeking behavior. In this study, a *p*-value of <0.05 (two-tailed) was considered statistically significant.

## Results

In this study, 1,243 adolescent girls in junior high schools participated, and 80.7% (1,003/1,243) of them had menstruation. The average age of the total adolescent girls and those with menstruation was 12.8 years (SD = 1.2) and 13.0 years (SD = 1.2), respectively. In this study, the number of adolescent girls in grade 6, 7, 8 and 9 were 348 (28.0%), 336 (27.0%), 269 (21.6%) and 290 (23.3%), respectively. In the 1,243 adolescent girls, over 42.3% of them had low weight, 70.0% of them were the only child in their family, 70.3% of their mothers and 67.0% of their fathers had an education of college and above, and 32.1% of them had higher level of family income. The proportion of adolescent girls with controllable learning burden and with uncontrollable learning burden were 79.9% and 8.9%, respectively. In this study, adolescent girls with menstruation tended to be older, had lower proportion of underweight, and had more learning burden issues Table 1.

**Table 1:** The demographic features of junior high school girls and their parents in Shanghai, China.

Variables	Total girls (*n* = 1,243)	Girls with menstruation (*n* = 1,003)	Girls without menstruation (*n* = 240)	t/*χ*^2^	*P*
Age (years), mean (SD)	12.8 (1.2)	13.0 (1.2)	11.6 (0.7)	23.17	<0.01
Grade, *n* (%)				247.88	<0.01
6	348 (28.0)	182 (52.3)	166 (47.7)		
7	336 (27.0)	276 (82.1)	60 (17.9)		
8	269 (21.6)	257 (95.5)	12 (4.5)		
9	290 (23.3)	288 (99.3)	2 (0.7)		
Height (cm), median (IQR)	161 (156–165)	162 (159–166)	154 (150–160)	232.72	<0.01
Weight (kg), median (IQR)	48.0 (42.55–55.0)	50.0 (45.0–55.0)	40.0 (35.0–46.0)	199.39	<0.01
BMI (kg/m2), n(%)				71.15	<0.01
<18 (low weight)	528 (42.3)	370 (70.1)	158 (29.9)		
18–24 (normal weight)	625 (50.3)	561 (89.8)	64 (10.2)		
≥24 (overweight/obesity)	90 (7.3)	72 (80.0)	18 (20.0)		
Nationality, *n* (%)				0.02	0.89
Han nationality	1,205 (96.9)	972 (80.7)	233 (19.3)		
Minority	38 (3.1)	31 (81.6)	7 (18.4)		
Siblings, *n* (%)				3.53	0.06
Yes	373 (30.0)	289 (77.5)	84 (22.5)		
No	870 (70.0)	714 (82.1)	156 (17.9)		
Education of mother, *n* (%)				3.72	0.29
Junior high or lower	73 (5.9)	61 (83.6)	12 (16.4)		
Senior high	120 (9.7)	101 (84.2)	19 (15.8)		
College and above	874 (70.3)	693 (79.3)	181 (20.7)		
No response	176 (14.2)	148 (84.1)	28 (15.9)		
Education of father, *n* (%)				8.54	0.04
Junior high or lower	52 (4.2)	42 (80.8)	10 (19.2)		
Senior high	142 (11.4)	123 (86.6)	19 (13.4)		
College and above	833 (67.0)	654 (78.5)	179 (21.5)		
No response	216 (17.4)	184 (85.2)	32 (14.8)		
Family income, *n* (%)				2.14	0.54
Higher level (>1.0 million RMB/year)	399 (32.1)	318 (79.7)	81 (20.3)		
Moderate level (0.2–1.0 million RMB/year)	501 (40.3)	413 (82.4)	88 (17.6)		
Lower level (<0.2 million RMB/year)	118 (9.5)	96 (81.4)	22 (18.6)		
No response	225 (18.1)	176 (78.2)	49 (21.8)		
Study burden condition, *n* (%)				5.81	0.05
Without learning burden	140 (11.3)	103 (73.6)	37 (26.4)		
With controllable learning burden	993 (79.9)	807 (81.3)	186 (18.7)		
With uncontrollable learning burden	110 (8.9)	93 (84.6)	17 (15.5)		

SD, standard deviation; IQR, interquartile range.

### Prevalence and factors associated with dysmenorrhea

**Figure 2:**
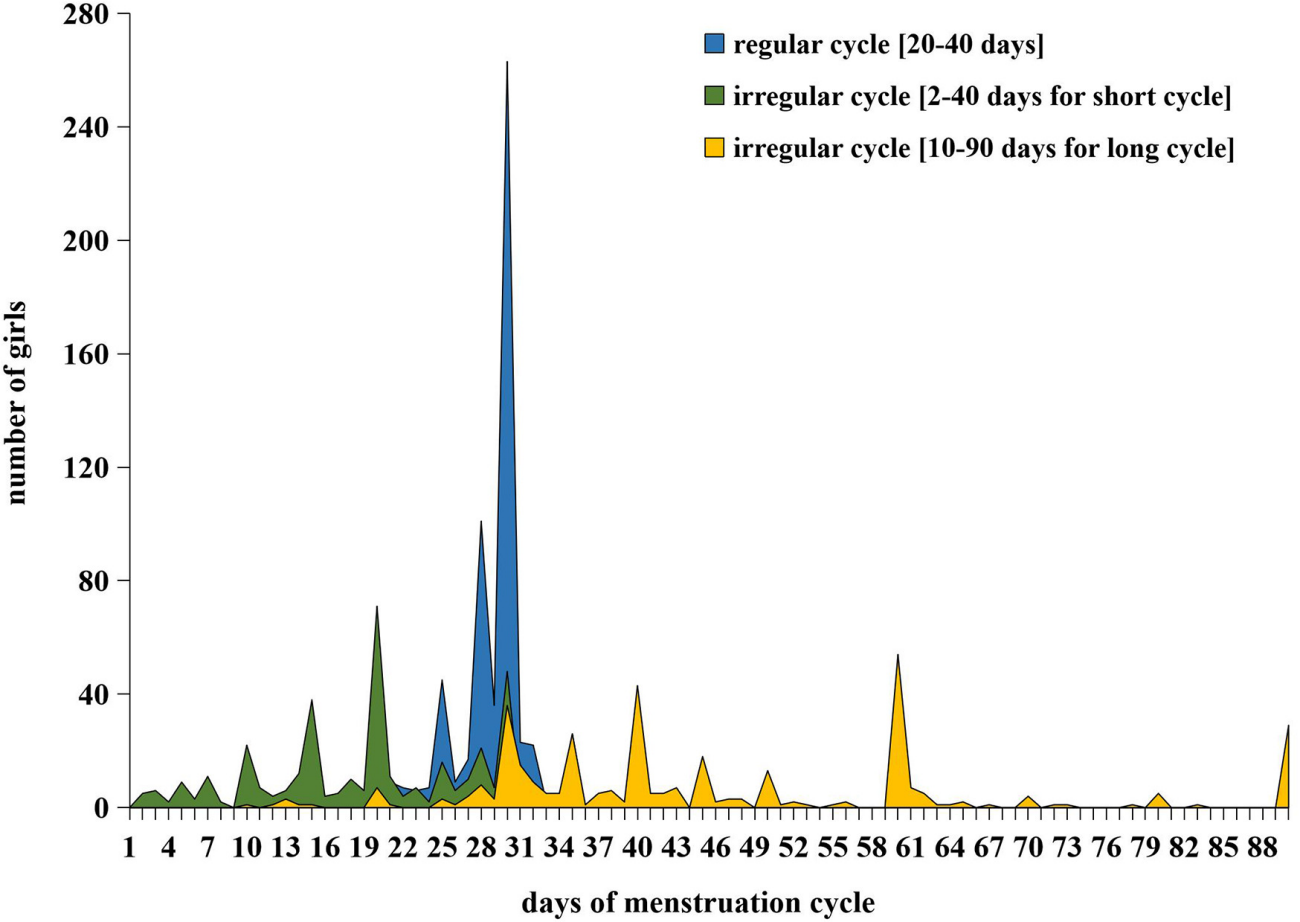
The days of menstruation cycle among junior high school girls with regular cycle and irregular cycle in Shanghai, China.

**Figure 3:**
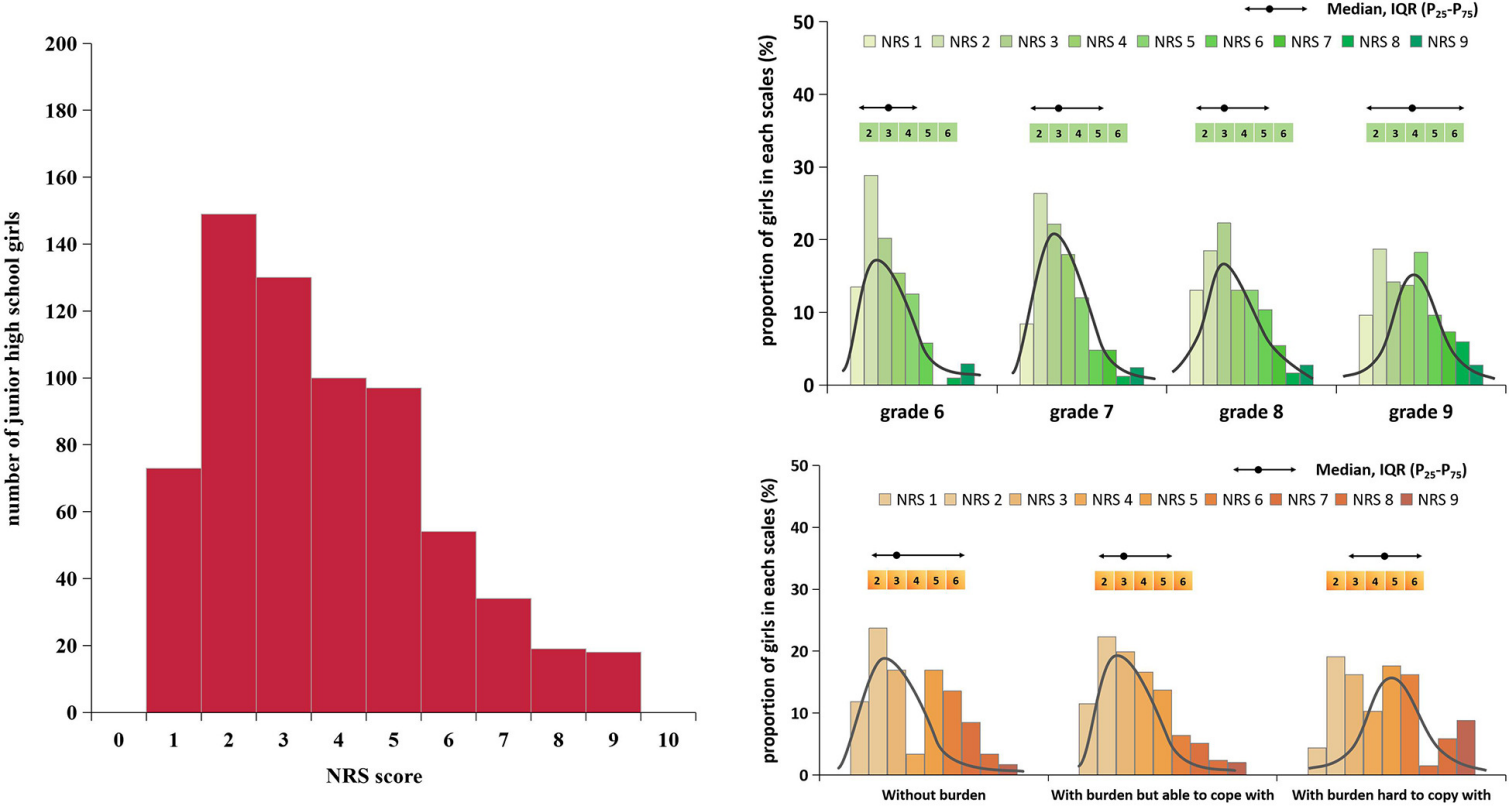
The NRS score of dysmenorrhea among junior high school girls by different grades and by different learning burden condition in Shanghai, China.

Among 1,003 adolescent girls with menstruation, the mean menarche age was 11.4 (SD = 0.9) years, 63.2% of them reported regular menstruation, and the mean menstrual cycle was 29.0 (SD = 3.9) days for those with a regular menstrual cycle, with a range of 20–40 days. The median menstrual cycle for adolescent girls with irregular menstruation was 22.0 days (IQR: 14.0–37.0), with a range of 2–40 days for short cycle and 10–90 days for long cycle. In this study, the prevalence of dysmenorrhea was 67.2%, and the median NRS score of dysmenorrhea severity was 3.0 (IQR: 2.0–5.0). In this study, adolescent girls in higher grade tended to have older menarche age, and the prevalence of regular menstruation was also higher among them. Moreover, adolescent girls in higher grade and with learning burdens had higher proportion of dysmenorrhea and with higher NRS score for dysmenorrhea severity, the differences were all statistically significant Table 2 and Figures 2,3.

**Table 2:** The menstrual period condition among junior high school girls with menstruation in Shanghai, China.

Variables	Total girls (*n* = 1,003)	Grade of girls				F/χ^2^	*P*
		Grade 6 (*n* = 182)	Grade 7 (*n* = 276)	Grade 8 (*n* = 257)	Grade 9 (*n* = 288)		
Menarche age (years), mean (SD)	11.4 (0.9)	10.9 (0.7)	11.3 (0.8)	11.5 (0.9)	11.8 (1.0)	42.63	<0.01
Duration of menstruation (days), median (IQR)	3 (3–3)	3 (3–3)	3 (3–3)	3 (3–3)	3 (3–3)	1.62	0.66
Menstruation condition, *n* (%)						14.36	<0.01
Regular menstruation	634 (63.2)	99 (54.4)	159 (57.6)	179 (69.6)	197 (68.4)		
Irregular menstruation	369 (36.8)	83 (45.6)	117 (42.4)	78 (30.4)	91 (31.6)		
Menstrual cycle for girls with regular period (days), mean (SD)	29.0 (3.9)	29.2 (3.2)	28.4 (4.0)	28.9 (3.9)	29.6 (4.1)	3.08	0.03
Cycle difference for girls with irregular period (days), median (IQR)	22.0 (14.0–37.0)	20.0 (11.0–37.0)	23.0 (14.0–33.0)	23.0 (15.0–46.0)	25.0 (15.0–35.0)	1.75	0.63
Sanitary napkins used in each period (packs), mean (SD)	3.0 (1.1)	2.9 (1.2)	2.8 (1.1)	3.1 (1.1)	3.1 (1.1)	4.84	<0.01
dysmenorrhea, *n* (%)						25.16	<0.01
Yes	674 (67.2)	104 (57.1)	167 (60.5)	184 (71.6)	219 (76.0)		
No	329 (32.8)	78 (42.9)	109 (69.5)	73 (28.4)	69 (24.0)		
NRS score of dysmenorrhea, median (IQR)^a^	3.0 (2.0–5.0)	3.0 (2.0–4.0)	3.0 (2.0–5.0)	3.0 (2.0–5.0)	4.0 (2.0–6.0)	16.90	<0.01
Absence of school due to dysmenorrhea, *n* (%)^a^						1.42	0.23
Yes	282 (41.8)	40 (38.5)	67 (40.1)	77 (41.9)	98 (44.8)		
No	392 (58.2)	64 (61.5)	100 (59.9)	107 (58.2)	121 (55.2)		
Hospital visit due to dysmenorrhea, *n* (%)^a^						0.19	0.66
Yes	82 (12.2)	13 (12.5)	20 (11.9)	18 (9.8)	31 (14.2)		
No	592 (87.8)	91 (87.5)	147 (88.1)	166 (90.2)	188 (85.8)		

SD, standard deviation; IQR, interquartile range.

^a^ The number of girls was 674, and was 104 in grade 6, 167 in grade 7, 184 in grade 8 and 219 in grade 9.

In this study, adolescent girls in grade 7 (OR = 1.15), grade 8 (OR = 1.89) and grade 9 (OR = 2.23) had higher prevalence of dysmenorrhea than those in grade 6, and adolescent girls with normal weight (OR = 1.38) or overweight/obesity (OR = 1.35) also had higher prevalence of dysmenorrhea. Moreover, adolescent girls whose mothers with education under college had higher prevalence of dysmenorrhea (OR was 1.87 for senior high, and 1.69 for junior high or lower), and adolescent girls with learning burden also had higher prevalence of dysmenorrhea (OR = 1.57 and 2.03) Table 3.

**Table 3:** Factors associated with the dysmenorrhea among junior high school girls in Shanghai, China.

Variables	Prevalence of dysmenorrhea, *n* (%)	Model A		Model B	
		OR	95% CI	OR	95% CI
Grade, *n* (%)‡					
6	104 (57.1)	1.00	–	1.00	–
7	167 (60.5)	1.15	0.79–1.68	1.18	0.80–1.74
8	184 (71.6)	**1**.**89**	**1.27–2.82**	**1**.**98**	**1.32–2.97**
9	219 (76.0)	**2**.**23**	**1.60–3.55**	**2**.**32**	**1.54–3.48**
BMI (kg/m2), *n* (%)‡					
<18 (low weight)	232 (62.7)	1.00	–	1.00	–
18–24 (normal weight)	392 (69.9)	**1**.**38**	**1.05–1.82**	1.29	0.97–1.71
≥24 (overweight/obesity)	50 (69.4)	1.35	0.79–2.33	1.05	0.60–1.83
Education of mother, *n* (%)‡					
Junior high or lower	46 (75.4)	1.69	0.92–3.09	1.77	0.95–3.27
Senior high	78 (77.2)	**1**.**87**	**1.14–3.05**	**1**.**85**	**1.12–3.07**
College and above	447 (64.5)	1.00	-	1.00	-
No response	103 (69.6)	1.26	0.86–1.85	1.27	0.86–1.88
Study burden condition, *n* (%)‡					
Without learning burden	59 (57.3)	1.00	–	1.00	–
With controllable learning burden	547 (67.8)	**1**.**57**	**1.03–2.38**	**1**.**69**	**1.10–2.60**
With uncontrollable learning burden	68 (73.1)	**2**.**03**	**1.11–3.70**	**2**.**04**	**1.10–3.80**
Nationality, *n* (%)					
Han nationality	656 (67.5)	1.00	–	–	–
Minority	18 (58.1)	0.67	0.32–1.38	–	–
Menarche age (years), *n* (%)				–	–
<11 years	102 (68.9)	1.00	–	–	–
≥11 years	572 (66.9)	0.91	0.63–1.33	–	–
Menstruation condition, *n* (%)				–	–
Regular menstruation	423 (66.7)	1.00	–	–	–
Irregular menstruation	251 (68.0)	1.06	0.81–1.40	–	–

Model A: Uni-variate logistic regression to explore factors associated the dysmenorrhea among junior high school girls (*n* = 1,003). Model B: Multi-variate logistic regression to explore factors associated the dysmenorrhea among junior high school girls (*n* = 1,003). OR, odds ratio; CI, confidence interval; BMI, body mass index.

Bold values are statistically significant (*p* < 0.05).

‡ The differences of dysmenorrhea prevalence between groups was statistically significant (*P* < 0.05).

Multivariable logistic regression analysis indicated that the prevalence of dysmenorrhea in grade 8 and grade 9 was 1.98 times (95% CI: 1.32–2.97) and 2.32 times (95% CI: 1.54–3.48) higher than that in grade 6 among adolescent girls in junior high schools. For adolescent girls whose mothers with senior high education had higher prevalence of dysmenorrhea (OR = 1.85, 95% CI:1.12–3.07) compared to adolescent girls whose mothers with an education of college and above. Adolescent girls experiencing learning burden demonstrated a significantly higher incidence of dysmenorrhea, the OR was 1.69 (95% CI: 1.10–2.60) for those with controllable learning burden and 2.04 (95% CI: 1.10–3.80) for those with uncontrollable burden, Table 3.

### Factors associated with school absenteeism in adolescent girls with dysmenorrhea

In this study, 41.8% adolescent girls with dysmenorrhea in junior high schools reported school absenteeism due to dysmenorrhea, and the percentage of school absenteeism increased gradually with the grade (38.5%-44.8%), with the highest percentage of 44.8% in grade 9. Table 4 indicated that adolescent girls with BMI < 18.5 had higher percentage of school absenteeism (50.4%), and those adolescent girls with learning burden also had higher percentage of school absenteeism (41.5% for controllable burden and 54.4% for uncontrollable burden). Moreover, adolescent girls with irregular menstruation had higher percentage of school absenteeism (50.6%) than those with regular menstruation Tables 2, 4.

**Table 4:** The factors associated school absenteeism and healthcare seeking experience due to dysmenorrhea in Shanghai, China.

Variables	Prevalence of school absenteeism, *n* (%)	Model 1		Prevalence of health care seeking, *n* (%)	Model 2	
		OR	95% CI		OR	95% CI
BMI (kg/m2), *n* (%)‡✝						
<18 (low weight)	117 (50.4)	1.00	–	38 (16.4)	1.00	–
18–24 (normal weight)	145 (36.9)	**0**.**60**	**0.43–0.83**	42 (10.7)	**0**.**60**	**0.37–0.97**
≥24 (overweight/obesity)	20 (40.0)	0.62	0.33–1.17	2 (4.0)	**0**.**19**	**0.04–0.83**
Education of mother, *n* (%)✝						
Junior high or lower	20 (43.5)	1.00	–	2 (4.4)	1.00	–
Senior high	31 (39.7)	0.89	0.42–1.90	14 (17.9)	**5**.**21**	**1.11–24.47**
College and above	183 (40.9)	0.90	0.48–1.70	50 (11.2)	2.65	0.62–11.39
No response	48 (46.6)	1.12	0.55–2.30	16 (15.5)	4.07	0.89–18.70
Study burden condition, *n* (%)‡						
Without learning burden	18 (30.5)	1.00	–	4 (6.8)	1.00	–
With controllable learning burden	227 (41.5)	1.74	0.96–3.14	70 (12.8)	2.17	0.75–6.24
With uncontrollable learning burden	37 (54.4)	**2**.**73**	**1.29–5.75**	8 (11.8)	1.95	0.55–6.95
Menstruation condition, *n* (%)‡✝						
Regular menstruation	155 (36.6)	1.00	–	43 (10.2)	1.00	–
Irregular menstruation	127 (50.6)	**1**.**74**	**1.26–2.39**	39 (15.5)	**1**.**60**	**1.00–2.57**

Model 1: Logistic regression to explore factors associated with the school absenteeism due to dysmenorrhea among junior high school girls (*n* = 674). Model 2: Logistic regression to explore factors associated with healthcare seeking experience due to dysmenorrhea among junior high school girls (*n* = 674). OR, odds ratio; CI, confidence interval.

Bold values are statistically significant (*p* < 0.05).

‡ The differences of school absenteeism prevalence between groups was statistically significant (*P* < 0.05).

✝ The differences of healthcare seeking experience prevalence between groups was statistically significant (*P* < 0.05).

Logistic regression indicated that adolescent girls with normal weight (OR = 0.60, 95% CI: 0.43–0.83) or overweight/obesity (OR = 0.62, 95%CI:0.33–1.17) had lower percentage of school absenteeism due to dysmenorrhea. Whereas, adolescent girls with controllable learning burdens (OR = 1.74, 95% CI: 0.96–3.14) or uncontrollable learning burden (OR = 2.73, 95% CI: 1.29–5.75) had higher percentage of school absenteeism. Adolescent girls in junior high schools with irregular menstruation had 1.74 times higher risk of school absenteeism compared to those with regular menstruation (95% CI: 1.26–2.39), Tables 2, 4.

### Factors associated with healthcare seeking behavior in adolescent girls with dysmenorrhea

In this study, 12.2% of the adolescent girls with dysmenorrhea in junior high schools had healthcare seeking experience due to dysmenorrhea. Table 4 indicated that adolescent girls with BMI < 18.5 had higher percentage of healthcare seeking behavior (16.4%), and adolescent girls whose mothers with education of senior high (17.9%) or college and above (11.2%) had higher percentage of healthcare seeking behavior due to dysmenorrhea. And adolescent girls with irregular menstruation had higher percentage of healthcare seeking experience (15.6%) than those with regular menstruation Tables 2, 4.

Logistic regression analysis indicated that adolescent girls with normal weight (OR = 0.60, 95% CI: 0.37–0.97) or overweight/obesity (OR = 0.19, 95%CI: 0.04–0.83) had less healthcare seeking experience due to dysmenorrhea. Adolescent girls whose mothers had senior high education were 5.21 (95%CI: 1.11–24.47) times more likely to have healthcare seeking experience, and adolescent girls with irregular menstruation had a 1.60 (95%CI: 1.00–2.57) times higher percentage of healthcare seeking experience Tables 2, 4.

## Discussion

This study finally recruited 1,243 adolescent girls in junior high schools in Shanghai, and the prevalence of dysmenorrhea was 67.2% among adolescent girls with menstruation. In this study, age of girls, BMI, academic burden, education of mother, and irregular menstruation were identified as factors associated with the dysmenorrhea, school absenteeism, and health care seeking behavior. Moreover, the academic stress (learning burden) was an important risk factor for dysmenorrhea as well as school absenteeism among adolescent girls.

Previous studies indicated that the prevalence of dysmenorrhea varied among adolescent girls due to its subjective perception feature and regional difference. This study reported a relatively high prevalence of dysmenorrhea among adolescent girls in junior high schools in Shanghai, which was in line with studies in Hong Kong (65.5%) (32), Southern Ethiopia (70%) (34), Malaysia (69.4%) (35), Singapore (83%) (36), and Australia (93%) (37), and as well as with a previously published systematic review (66.1%) (38). However, the dysmenorrhea prevalence in this study was significantly higher than female university students in Changsha (41.7%) (26). These variation in dysmenorrhea prevalence might be due to the differences in age, social-cultural status, and different pain perception. Nevertheless, the lack of a standardized definition of dysmenorrhea could also partially explain the variation of dysmenorrhea prevalence in different studies.

In this study, the prevalence of dysmenorrhea increased with grade among adolescent girls in junior high school from 57.1% for grade 6 to 76.0% for grade 9, which was in line with the findings in a study implemented in Japan (39). For primary dysmenorrhea in adolescent girls, it was mainly caused by the increased prostaglandin levels in menstrual blood; anovulatory endometrium was less likely to induce dysmenorrhea because of the low concentration of prostaglandin. So, dysmenorrhea usually occurs in the ovulatory cycle among young girls due to anovulatory endometrium is not established immediately after the first menstrual period (40). Therefore, adolescent girls in higher grade with older age tended to establish a stable ovulatory cycle, and have an increased likelihood of dysmenorrhea.

Our study showed that adolescent girls with low BMI had a lower prevalence of dysmenorrhea. These results are compatible with other research findings (41, 42). However, the association between BMI and PD is still controversial. Many studies have revealed no relationship between BMI and dysmenorrhea (43, 44), whereas other studies have shown an increased prevalence of PD in low BMI subjects (45, 46). Despite that the pathophysiological mechanisms are still unclear, a possible hypothesis is that a lower amount of body fat affects normal ovulation and menstrual cycles and thus leads to excessive release of prostaglandin (PGs); higher circulating levels of PGs have been reported in women with dysmenorrhea compared with asymptomatic women during menstruation (47). Recent evidence has highlighted the role of systemic inflammatory conditions in the pathophysiology of dysmenorrhea. Notably, a 2025 study (48) demonstrated that adolescent girls with Familial Mediterranean Fever (FMF) experienced significantly more frequent and severe dysmenorrhea compared to healthy controls. Underlying inflammatory disorders such as familial Mediterranean fever (FMF) can contribute to dysmenorrhea through multiple pathological mechanisms. Primarily, these conditions stimulate excessive production of inflammatory mediators (e.g., IL-1β, TNF-ɑ), which directly sensitize uterine pain pathways. Furthermore, they dysregulate prostaglandin biosynthesis, resulting in pathological elevations of uterine spasm. Additionally, chronic inflammation may induce estrogen level fluctuations that alter uterine contractility patterns and pain perception thresholds. Dysmenorrhea can be a stressor and aggravate the symptoms of depression and anxiety (49), In a previous systematic review, Latthe et al. found that women with dysmenorrhea had 2.77 times more chance of experiencing anxiety and 2.59 times higher chance of depression (50). Another recent systematic review by Bajalan et al. showed the possible association between primary dysmenorrhea and depression/anxiety (51). Psychological disorders such as depression, stress, and anxiety are reported as important factors associated with dysmenorrhea and menstrual disorders (52–54). Dysmenorrhea can also impact negatively on daily activities, lower education performance at puberty, and lead to poor sleep quality, and have negative effects on mood resulting in anxiety and depression (55–57).

In addition, this study indicated that learning burden was positively correlated to the prevalence of dysmenorrhea among adolescent girls, which was also reported in previous study (58). The stress experienced by girls can impact the function of endocrine system, which could increase the prostaglandin secretion, lead to excessive uterine contractions, and then cause dysmenorrhea (59). Dysmenorrhea is a major cause for school absenteeism in adolescent girls at school age. In this study, 41.8% of adolescent girls in junior high schools reported school absenteeism due to dysmenorrhea. A national survey in Bangladesh found that 41% of menstruating girls aged 11–17 years reported school absenteeism during menstruation (60). A study of high school girls in Kuwait found that 58.2% of students with dysmenorrhea missed at least one day of school (61). In Nigeria, a cross-sectional study enrolled 583 female university students found that 43% of them reported school absenteeism due to dysmenorrhea (62). So, we could notice that the dysmenorrhea could significantly disadvantage girls in their studies globally, even among university students. Therefore, education officials should interact with health officials to promote health education focusing on dysmenorrhea and menstruation among adolescent girls and their parents, and provide medical as well as academic assistance for adolescent girls during their school absenteeism.

In this study, we noticed that the prevalence of school absenteeism increased with grades among adolescent girls in junior high schools, this might due to the fact that adolescent girls in higher grade tend to have a higher NRS score of dysmenorrhea severity, which might lead to the increased number of school absenteeism. Finding in this study was in line with a study implemented in Australian which also indicating that higher pain scores were positively correlated with more frequent absenteeism from class (63). In addition, this study indicated that academic stress and irregular menstruation were risk factors for school absenteeism among adolescent girls. Previous studies demonstrated that high academic stress could lead to endocrine disruption and increase the risk of dysmenorrhea, and irregular menstruation was also a risk factor of dysmenorrhea (64). So we should advocate adolescent girls to keep a healthy lifestyle and adjust their emotions promptly to avoid excessive stress. Nevertheless, school officials and health program coordinators might benefit from adolescent girls during their school absenteeism by providing academic assistance and reducing academic stress.

In this study, only 31.1% of adolescent girls in junior high schools with dysmenorrhea had healthcare seeking experience, however the proportion of healthcare seeking in Chinese adolescent girls was higher than Hispanic female adolescents (14%) (27). In addition, this study indicated that adolescent girls with low body weight had higher proportion of health care seeking experience which might due to the fact that adolescent girls with low body weight prone to have higher NRS score for dysmenorrhea severity. This study indicated that adolescent girls whose mother had junior high education or lower were less likely to seek healthcare service, because the primary information about menstruation and dysmenorrhea among adolescent girls was usually acquired from their mothers (65). Mothers with lower education usually had inadequate information about menstrual physiology and with more conservative social attitudes, so they were less like to advise their daughters to seek healthcare assistance when encountering dysmenorrhea.

This study demonstrated that dysmenorrhea was an emerging serious gynecological and public health issue among adolescent girls, and highlighted the necessity of providing targeted intervention and prevention measures. We recommend establishing interdisciplinary collaborative mechanisms among schools, healthcare providers, and policymakers to deal with the dysmenorrhea among adolescent girls, especially among those with under body weight, those whose mothers with low education, and those with irregular menstruation and academic stress. Firstly, we suggest that systematic health education courses be offered at the school level to eliminate students’ cognitive misunderstandings and shame about menstruation. Meanwhile, schools need to build up the capacity of teachers and provide resource support. For instance, they should collaborate with medical experts to conduct qualification training for teachers to ensure that they master scientific knowledge about menstrual health and communication skills. It is also possible to set up campus health corners in schools equipped with emergency kits for hygiene products, science popularization brochures and other supplies, and ensure the accessibility of these supplies. Secondly, we suggest that healthcare providers can offer professional consultation and clinical services, such as conducting regular on-site consultations in schools and providing personalized guidance for common problems like dysmenorrhea and menstrual disorders (such as hot compress methods and drug usage norms); At the same time, a standardized health management plan should be established, such as formulating the “Guidelines for Menstrual Health Management during Adolescence”, clearly defining non-pharmaceutical intervention measures such as dietary regulation and exercise suggestions (such as low-intensity yoga during menstruation). Thirdly, we suggest that policymakers incorporate menstrual health education into the compulsory education curriculum, stipulate minimum class hour standards and teaching quality assessment mechanisms, and at the same time promote the establishment of a collaboration mechanism between public hospitals and schools, clarifying the responsibility boundaries of medical teams in campus health services. In addition, jointly issued the “Menstrual Health Campus Action Guide” with the education and health departments, clarifying the multi-party collaboration process and data sharing mechanism; It is also possible to encourage the media to carry out popular science publicity and eliminate the tendency of stigmatizing menstrual issues in social culture. We anticipate that the implementation of the aforementioned intervention measures will relieve their menstruation worry, reduce school absenteeism and increase healthcare seeking behavior, among finally improve physical and mental health level among adolescent girls.

## Strengths and limitations

Strength of this study is the integrated exploration of the prevalence and factors associated with dysmenorrhea, school absenteeism, and healthcare seeking behavior among adolescent girls at school age. However, this study has several limitations. First, adolescent girls in study were enrolled from 5 junior high schools in Hongkou district in Shanghai, which ensures the high internal authenticity, however, the generalization of the findings to represent the whole story in Shanghai is limited. Second, the nature of cross sectional study could only provide the primary association between the academic stress and dysmenorrhea, school absenteeism and healthcare seeking among adolescent girls, but could not establish the causal relationship. Third, it was difficult to differentiate the primary and secondary dysmenorrhea, and some adolescent girls might under reporting their school absenteeism experience, all of which could induce information bias in this study. Therefore, incorporating improvements should be considered in future studies. Fourth, this study temporarily adopts the adult BMI classification criteria for participants under 18 years of age. This approach is primarily implemented because the survey simultaneously includes individuals aged 18 and above. To maintain longitudinal data comparability and prevent analytical discontinuities caused by switching criteria, we standardized the BMI classification method. However, this may introduce certain deviations in the BMI categorization within this study. Additionally, the height and weight used for BMI calculation in this study were collected from self-reported questionnaire interview, which might induce reporting bias.

## Conclusion

This study reveals a substantial prevalence of dysmenorrhea, which correlates with grade level, academic stress, maternal education, and irregular menstruation. It underscores the urgent necessity for heightened attention and targeted interventions concerning menstrual health among adolescent girls in junior high schools. The research findings furnish scientific substantiation for refining and enforcing public health policies aimed at enhancing the health and well-being of adolescent girls.

## Data Availability

The raw data supporting the conclusions of this article will be made available by the authors, without undue reservation.
